# Effects of Organochlorine Contaminants on Loggerhead Sea Turtle Immunity: Comparison of a Correlative Field Study and *In Vitro* Exposure Experiments

**DOI:** 10.1289/ehp.8143

**Published:** 2005-09-21

**Authors:** Jennifer M. Keller, Patricia D. McClellan-Green, John R. Kucklick, Deborah E. Keil, Margie M. Peden-Adams

**Affiliations:** 1 Nicholas School of the Environment and Earth Sciences, Coastal Systems Science and Policy, and Integrated Toxicology Program, Duke University, Beaufort, North Carolina, USA; 2 National Institute of Standards and Technology, Hollings Marine Laboratory, Charleston, South Carolina, USA; 3 Department of Environmental and Molecular Toxicology, and Center for Marine Science and Technologies, North Carolina State University, Morehead City, North Carolina, USA; 4 Marine Biomedicine and Environmental Science Center, and; 5 Department of Pediatrics, Medical University of South Carolina, Charleston, South Carolina, USA; 6 Grice Marine Laboratory, College of Charleston, Charleston, South Carolina, USA; 7 Mystic Aquarium and Institute for Exploration, Mystic, Connecticut, USA

**Keywords:** DDT, immunotoxicity, organochlorine contaminants, organochlorine pesticides, PCBs, persistent organic pollutants, polychlorinated biphenyls, reptile

## Abstract

Several laboratory and field studies indicate that organochlorine contaminants (OCs), such as poly-chlorinated biphenyls (PCBs) and pesticides, modulate immune responses in rodents, wildlife, and humans. In the present study we examined the effects of OCs on immunity in free-ranging loggerhead sea turtles (*Caretta caretta*). Mitogen-induced lymphocyte proliferation responses, lysozyme activity, and OC concentrations were measured from blood samples. Mitogens chosen in the lymphocyte proliferation assay were phytohemagglutinin (PHA) and concanavalin A (ConA) for T-lymphocyte stimulation, and lipopolysaccharide (LPS) and phorbol 12,13-dibutyrate (PDB) for B-lymphocyte stimulation. Lysozyme activity was significantly and negatively correlated with whole-blood concentrations of 4,4′-dichlorodiphenyldichloroethylene (4,4′-DDE) and the sum of chlordanes. Lymphocyte proliferation responses stimulated by PHA, LPS, and PDB were significantly and positively correlated with concentrations of the sum of PCBs measured in whole blood. LPS- and PDB-induced proliferation were also significantly and positively correlated with 4,4′-DDE blood concentrations. These correlative observations in free-ranging turtles suggest that current, chronic exposure to OCs may suppress innate immunity and enhance certain lymphocyte functions of loggerhead sea turtles. To further test this hypothesis, lymphocyte proliferation was measured after *in vitro* exposure of peripheral blood leukocytes from 16 turtles to Aroclor 1254 (0–13.5 μg/mL) or 4,4′-DDE (0–13.4 μg/mL). Both contaminants increased PHA- and PDB-induced proliferation at concentrations below those that affected cell viability. Moreover, the concentrations that enhanced PDB-induced proliferation *in vitro* were similar to concentrations measured in turtles with the highest proliferative responses. The similarities between the *in vitro* experiments and the correlative field study suggest that OC exposure modulates immunity in loggerhead turtles.

Environmental contaminants, such as organochlorine contaminants (OCs), have been shown to affect the immune functions of animals exposed in the laboratory (Harper et al. 1993; Ross et al. 1996; Segre et al. 2002; Silkworth et al. 1984; Smialowicz et al. 1989; Smits et al. 2002; Wu et al. 1999). These experiments have substantiated relationships observed between OC concentrations and immunomodulation in free-ranging wildlife (Grasman and Fox 2001; Lahvis et al. 1995; reviewed by Keller et al. 2000). OCs, such as polychlorinated biphenyls (PCBs), 4,4′-dichlorodiphenyldichloroethylene (4,4′-DDE), chlordanes, and other pesticides, have been recently documented in blood and adipose tissue of loggerhead sea turtles (*Caretta caretta*) from North Carolina (Keller et al. 2004a). Although the concentrations were low relative to other wildlife species that feed at higher trophic levels, the concentrations significantly correlated with several health indicators, including white blood cell counts and some plasma chemistry measurements (Keller et al. 2004c). The effects of environmental contaminants on functional aspects of sea turtle immunity, however, have not yet been addressed in any published study.

Sea turtles face many impacts from human activity, including hunting, fisheries interactions, loss of nesting habitat due to coastal development, and anthropogenic chemical contamination. Of these threats, the effects of environmental contaminants on sea turtle health are the least understood, and few studies have addressed this potential impact (Aguirre et al. 1994; Day 2003; Heesemann et al. 2004; Keller et al. 2004c; Lutcavage et al. 1995; Peden-Adams et al. 2002, 2003; Podreka et al. 1998). All species of sea turtles found in U.S. waters are protected by the U.S. Endangered Species Act under either an endangered or threatened status (Pritchard 1997). Specifically, the loggerhead sea turtle (*Caretta caretta*) is protected as a threatened species, and although some populations are recovering, others may still be declining (Turtle Expert Working Group 2000). It is therefore important to understand the risk that contaminants pose to the general health and immunologic function of loggerhead sea turtles because these effects could affect the survival of their populations.

In this study we examined how OCs influence loggerhead sea turtle immune responses using the mitogen-induced lymphocyte proliferation assay and plasma lysozyme activity. The lymphocyte proliferation assay has been optimized for loggerhead and green sea turtles (*Chelonia mydas*) (Keller et al. 2005; McKinney and Bentley 1985; Work et al. 2000). Lysozyme activity, a measure of innate immunity, has not previously been reported for any sea turtle species. Because lymphocyte proliferation and lysozyme activity have been shown to be altered by OC exposure in other species (Burton et al. 2002; Grasman and Fox 2001; Lahvis et al. 1995; Ross et al. 1996; Segre et al. 2002; Smits et al. 2002; Wu et al. 1999), we hypothesized that OC exposure may also modulate these immune functions in loggerhead sea turtles. If shown to be affected by OCs, these immune measurements would offer a relatively simple biomarker that requires only a nonlethal blood sample.

## Materials and Methods

### Sampling.

All turtles used in this study were treated humanely in accordance with protocols approved by Duke University Institutional Animal Care and Use Committee (protocols A351-99-07-2, A351-99-07-3, and A206-01-07) and with required federal and state permits (U.S. Fish and Wildlife Service permits PRT-676379 and TE-676379-2, National Marine Fisheries Service permit 1245, and North Carolina Wildlife Resources Commission permits 00ST70 and 01ST45). Forty-eight free-ranging juvenile loggerhead sea turtles with straight carapace lengths (SCLs; measured from the nuchal notch to the most posterior marginal notch) between 45.7 and 77.3 cm were captured as by-catch from a pound net fishery located in Core Sound, North Carolina, in July and August 2000 and in July 2001. Most turtles appeared healthy upon visual examination, and body condition indices and plasma chemistry values were measured and reported elsewhere (Keller et al. 2004c). Lymphocyte proliferation was measured in only the 2001 samples, whereas lysozyme activity was measured in both years. Blood was collected within 10 min of capture from the dorsocervical sinus using double-ended Vacutainer needles directly into Vacutainer blood collection tubes containing sodium heparin (Becton Dickinson, Franklin Lakes, NJ) and kept cool until processing. One blood tube from each turtle was frozen at −20°C for contaminant analysis. Plasma from another blood tube collected from 45 of the turtles was frozen at −80°C for lysozyme activity measurements. An additional blood tube from 24 of the turtles captured only in July 2001 was processed for lymphocyte proliferation. Turtles were tagged, measured, weighed, and released near their capture location. Body condition was calculated as weight (kilograms) divided by the cube of SCL (centimeters) and multiplied by 100,000 [body condition = weight/(SCL^3^) × 100,000] as described by Bjorndal et al. (2000). Sex of the turtles was determined by measuring plasma testosterone concentrations (Owens 1997). An additional 16 juvenile turtles (SCL ranged from 52.8 to 72.3 cm) were captured in offshore waters of South Carolina, Georgia, and northeastern Florida during June 2003 (*n* = 8) and June 2004 (*n* = 8). These turtles were randomly captured in trawl nets without turtle excluder devices at randomly selected stations using a trawl tow time of 30 min. Blood samples were collected and processed in the same manner as described above for use in *in vitro* exposure experiments.

### Contaminant analysis.

Concentrations of OCs, including 55 PCB congeners and 24 pesticides, were determined in whole blood of the North Carolina turtles and are reported elsewhere (Keller et al. 2004a). Briefly, samples were amended with internal standards and extracted with organic solvents. After lipid content was determined gravimetrically, biologic molecules of large molecular weight were removed from the extracts using alumina columns. PCBs were separated from the pesticides by polarity into two fractions using an aminopropylsilane column. Compounds were quantified using gas chromatography with electron capture and mass spectrometry detection. Analytical blanks and standard reference materials from the National Institute of Standards and Technology were analyzed with each batch of samples. The blood lipid content did not correlate to blood OC concentrations (Keller JM, unpublished data); therefore, the blood concentrations were calculated based on the wet mass of blood extracted (nanograms per gram wet mass). OC concentrations that were below the detection limit were estimated at half the detection limit for correlations. The detection limits were calculated as the amount (nanograms) of compound in the most dilute calibration standard solution yielding a signal significantly different from the noise, divided by the grams of tissue extracted.

### Lysozyme activity.

We measured lysozyme activity using slight modifications of a standard turbidity assay as previously described by Demers and Bayne (1997). A 1 mg/mL stock solution of hen egg lysozyme (HEL; Sigma, St. Louis, MO) was prepared in 0.1 M phosphate buffer (pH 5.9), and aliquots were frozen until use. A solution of *Micrococcus lysodeikticus* (Sigma) was prepared fresh daily by dissolving 50 mg of the lyophilized cells in 100 mL 0.1 M phosphate buffer (pH 5.9). HEL was serially diluted in phosphate buffer to produce a standard curve of 40, 20, 10, 5, 2.5, 1.25, 0.6, 0.3, and 0 μg/μL. Aliquots of each concentration (25 μL/well) were added to a 96-well plate in triplicate. For each sample, 25 μL of test plasma was added in quadruplicate to the plate. The solution of *M. lysodeikticus* (175 μL/well) was quickly added to three sample wells and to each of the standard wells. The fourth well containing plasma received 175 μL phosphate buffer and served as a blank. Plates were assessed for absorbance at 450 nm with a spectrophotometer (SpectraCount; Packard, Meridian, CT) immediately (T0) and again after 5 min (T5). Absorbance unit (AU) values at T5 were subtracted from AU values at T0 to determine the change in absorbance. The AU value for the blank sample well was subtracted from the average of the triplicate sample wells to compensate for any hemolysis in the samples. The resultant AU value was converted to HEL concentration (micrograms per microliter) via linear regression of the standard curve.

### Mitogen-induced lymphocyte proliferation.

#### Lymphocyte proliferation assay for correlations with OCs.

Lymphocyte proliferation responses have been reported elsewhere (Keller et al. 2005). Rather than a density gradient method, peripheral blood leukocytes (PBLs) were collected from the buffy layer within 36 hr of blood collection using a slow-spin technique (42 × *g* for 25 min) as described in detail by Keller et al. (2005). No density gradient method is available to obtain a pure isolation of loggerhead lymphocytes (Harms et al. 2000). Cells were rinsed once with cell culture media composed of RPMI 1640 media (Mediatech, Inc., Herndon, VA) supplemented with final concentrations of 5% fetal bovine serum (FBS; Hyclone, Logan, UT), 1% (vol/vol) 100× solution of nonessential amino acids (Gibco, Grand Island, NY), 1 mM sodium pyruvate (Gibco), 10 mM HEPES (Mediatech), 50 IU/mL penicillin, and 50 μg/mL streptomycin (Mediatech) and initially brought to pH 6.9.

Viable PBLs were counted by trypan blue exclusion using light microscopy. Although this technique cannot distinguish sea turtle lymphocytes from thrombocytes or other small PBLs (Work et al. 1998), thrombocytes are known to aggregate. The aggregating cells were not counted in order to decrease the chance of counting these nontarget cells. There is no evidence in the literature that thrombocytes would proliferate in the presence of a mitogen; furthermore the use of a stimulation index (SI) should account for any potential, although unexpected, background proliferation of any other PBL type.

We split the cell suspension into two tubes and diluted in two different media compositions; media 1, as described above, or media 2, which differed only by the FBS manufacturer (BioWhittaker, Walkersville, MD). Cells were plated at 1.8 × 10^5^ cells/well into 96-well plates. We used phytohemagglutinin P (PHA) and concanavalin A (ConA) as T-lymphocyte mitogens, and lipopolysaccharide (LPS) and phorbol 12,13-dibutyrate (PDB) as B-lymphocyte mitogens. PDB has previously been shown to stimulate avian B lymphocytes (Scott and Savage 1996). ConA from Jack bean type IV-S (C5275, Sigma) and LPS from *Escherichia coli* serotype 0111:B4 (L2630, Sigma) were diluted in media 1. PHA (Amersham Pharmacia Biotech Inc., Pascataway, NJ), ConA type IV from jack bean (*Canavalia ensiformis*) (C2010, Sigma), and LPS from *E. coli* serotype 0127:B8 (L3129, Sigma) were diluted in media 2. PDB (Sigma) was tested in both media types. Cells were tested in triplicate for each unstimulated control (containing only media 1 or 2, respectively) and each mitogen concentration with final volumes at 200 μL/well for mitogens in media 1 and 100 μL/well for mitogens in media 2. The plates were incubated at 30°C with 5% CO_2_. Final concentrations of mitogens in culture wells and culture conditions are listed in Table 1 and in each figure.

We tested proliferation by adding 0.5 μCi ^3^H-thymidine (ICN Biomedical, Irvine, CA) in a volume of 100 μL to each well after a 4-day incubation (96 hr) or a 5-day incubation (120 hr) with mitogens. Plates were further incubated for an additional 16 hr and then harvested onto Unifilter plates (Packard, Meridian, CT) using a Packard Filtermate 96-well plate harvester. The plates were allowed to dry, and 25 μL Microscint 20 (Packard) was added to each well. The samples were analyzed using a Packard Top Count-NXT scintillation counter. Unstimulated wells produced counts per minute (cpm) that ranged from 20 cpm to 1,162 cpm. Only three samples produced < 300 cpm. The SI was calculated as the counts per minute of mitogen-stimulated cells divided by the counts per minute of unstimulated control (media only) cells. Not all mitogen concentrations and stimulation durations were tested on all available samples because the samples collected during the 2001 sampling season were also used to optimize the lymphocyte proliferation assay. Decisions regarding optimal conditions were not yet determined, and thus the sample sizes vary among the different culture conditions (Table 1).

#### Lymphocyte proliferation assay for *in vitro* exposure experiments.

PBLs from 16 loggerhead turtles (8 captured in 2003 and 8 captured in 2004) were collected as described above. Cells and mitogens were diluted in a third media composition identical to media 1 described above except for the FBS manufacturer (Gemini, Calabasas, CA). This change in FBS source was shown in paired samples in 2003 not to alter the lymphocyte proliferation response (Keller et al. 2005). Cells were plated at 1.8 × 10^5^ cells/well in a final volume of 200 μL/well. Final mitogen concentrations in the culture wells were 0.2 μg/mL PDB (Sigma) or 5 μg/mL PHA (Amersham Pharmacia Biotech in 2003; L9132 from Sigma in 2004). Paired sample experiments with these two PHA sources indicated no difference in the proliferation response (Peden-Adams MM, unpublished data).

In 2003, solutions of 100 μg/mL Aroclor 1254 and 4,4′-DDE (both from ChemService, West Chester, PA) in methanol were evaporated to dryness under a stream of ultra-high-purity nitrogen and dissolved into dimethyl sulfoxide (DMSO). In 2004, neat Aroclor 1254 (Supelco, Bellefonte, PA) and 4,4′-DDE (Aldrich Chemical Co., Milwaukee, WI) were weighed and diluted in sterile DMSO to make stock solutions. The stocks were further diluted in media, and 5 μL/well of these substocks was added after plating the cells. The final concentration of DMSO in each culture well, including the DMSO control wells, was standardized to 0.03% (vol/vol). Non-DMSO controls, DMSO controls, and each contaminant concentration were tested in triplicate with and without mitogen stimulation for each turtle. The concentrations of Aroclor 1254 (a technical mixture of PCBs) in the culture wells were 0, 0.1, 1.0, 2.5, 5.0, 10, 48, 100, 498, 993, 7,500, and 13,500 ng/mL, and the concentrations of 4,4′-DDE in the culture wells were 0, 0.05, 0.1, 0.25, 0.5, 0.75, 1.0, 5.0, 48, 500, 992, 7,500, and 13,400 ng/mL. PBLs from individual turtles were exposed to all concentrations, resulting in a dose–response relationship for each individual animal. Cells were incubated at 30°C with 5% CO_2_ for 5 days, at which time ^3^H-thymidine was added and proliferation was measured 16 hr later, as described above. Cell viability was determined by trypan blue exclusion of cells from an additional nonstimulated replicate of each contaminant concentration on day 5 of the exposure.

The blood OC concentrations were not measured from these 16 turtles, but the PBLs were rinsed free of plasma where most of the OCs distribute in loggerhead sea turtle blood (Keller et al. 2004b). Because prior exposure may affect a turtle’s cellular response to the *in vitro* exposure, the data were handled to reduce interindividual variability. The response of each turtle at each contaminant concentration was calculated as a percentage of its non-DMSO control response. For example, the exposed SI for 5 ng/mL 4,4′-DDE was calculated as counts per minute of cells exposed to PHA plus 5 ng/mL 4,4′-DDE divided by counts per minute of cells exposed to only 5 ng/mL 4,4′-DDE. This SI value was then normalized to the turtle’s non-DMSO SI (counts per minute of cells exposed to PHA divided by counts per minute of cells exposed to only media).

### Statistics.

We used nonparametric Spearman rank correlations because contaminant concentrations did not fit a normal distribution before or after transformation. We used these correlations to examine the relationship of lymphocyte proliferation and lysozyme activity with OC concentrations determined in the blood (nanograms per gram wet mass). The proportional responses of the *in vitro* exposure experiments were not normally distributed, so the data were log-transformed. Analysis of variance (ANOVA) with a Dunnet’s multiple comparison test was used to compare the responses at each contaminant concentration to the DMSO control. All statistical analyses were performed using JMP 4.0.2 (SAS Institute Inc., Cary, NC).

## Results

### Organochlorine concentrations.

OC concentrations in samples from turtles captured in 2000 and 2001 have been reported elsewhere on both a wet-mass and a lipid-normalized basis (Keller et al. 2004a). For the purpose of comparing immune function and OC concentrations, only samples from turtles assessed for lymphocyte proliferation in July 2001 are described here (*n* = 27). The mean (± SE) concentrations of ∑PCBs (the sum of PCBs), 4,4′-DDE, and ∑chlordanes (the sum of chlordanes) were 6.25 ± 1.14 ng/g wet mass, 0.721 ± 0.152 ng/g wet mass, and 0.253 ± 0.048 ng/g wet mass, respectively. The concentrations of these compounds in this group of turtles were not significantly different from turtles sampled in 2000 (Mann-Whitney *t*-test; all *p*-values > 0.05).

### Plasma lysozyme activity.

**Figure 1:**
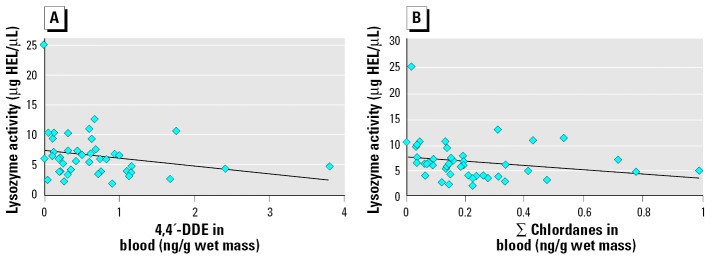
Scatterplots of plasma lysozyme activity versus concentrations of 4,4′-DDE (*A; r*_S_ = −0.310, *p* = 0.038) and ∑chlordanes (*B; r*_S_ = −0.368, *p* = 0.013) measured in the blood of loggerhead sea turtles. Linear trend lines demonstrate the negative relationships determined using Spearman rank correlations.

We measured lysozyme activity in plasma samples from 45 animals that were assessed for OC concentrations. Lysozyme activity was 6.58 ± 0.58 μg HEL/μL (mean ± SE) with a range of 1.94–25.2 μg HEL/μL. The Spearman correlation coefficients, *r*_S_ (*p*-values) for lysozyme versus ∑PCBs, 4,4′-DDE, and ∑chlordanes were −0.269 (0.074), −0.310 (0.038), and −0.368 (0.013), respectively. All slopes were negative, and correlations with 4,4′-DDE and ∑chlordanes were statistically significant at α = 0.05 (Figure 1). Lysozyme activity did not differ between gender and did not correlate to body condition or plasma testosterone levels.

### Lymphocyte proliferation: correlations with blood OC concentrations.

The mean lymphocyte proliferation responses, reported elsewhere (Keller et al. 2005), are tabulated along with the correlative results between lymphocyte proliferation and OC concentrations in Table 1. Proliferation in this data set did not differ between sexes and did not correlate with testosterone concentrations (data not shown). Body condition correlated with lymphocyte proliferation only in media type 1 when stimulated with 0.2 μg/mL PDB for 5 days (*r*_S_ = 0.528; *p* = 0.012).

**Figure 2:**
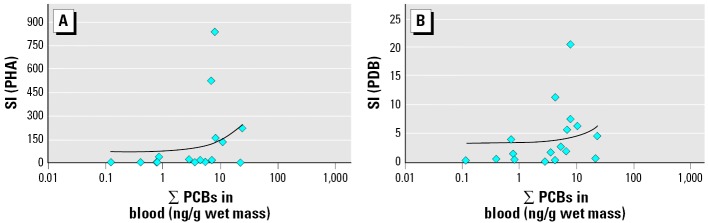
Scatterplots of ∑PCB blood concentrations versus loggerhead sea turtle lymphocyte proliferation (SI) responses stimulated for 5 days with 5 μg/mL PHA (*A; r*_S_ = 0.596, *p* = 0.012) and 0.8 μg/mL PDB (*B; r*_S_ = 0.564, *p* = 0.018). SI = cpm of mitogen-stimulated cells/cpm of unstimulated cells. Linear trend lines demonstrate the positive relationships determined using Spearman rank correlations.

**Figure 3:**
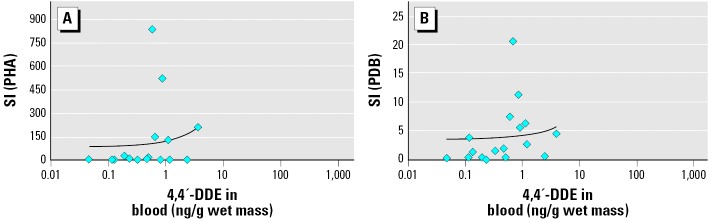
Scatterplots of 4,4′-DDE blood concentrations versus loggerhead sea turtle lymphocyte proliferation (SI) responses stimulated for 5 days with 5 μg/mL PHA (*A; r*_S_ = 0.431; *p* = 0.084) and 0.8 μg/mL PDB (*B; r*_S_ = 0.507; *p* = 0.038). SI = cpm of mitogen-stimulated cells/cpm of unstimulated cells. Linear trend lines demonstrate the positive relationships determined using Spearman rank correlations.

Some proliferative responses were significantly correlated with blood OC concentrations. Lymphocyte proliferation stimulated by 4 days of exposure to 10 μg/mL LPS was significantly correlated with blood concentrations of ∑PCBs, 4,4′-DDE, and ∑chlordanes. PHA-induced proliferation (5 μg/mL PHA) correlated with blood concentrations of ∑PCBs. Proliferation stimulated by 0.8 μg/mL PDB was also positively correlated with ∑PCB and 4,4′-DDE concentrations. ConA stimulation, however, did not correlate with any contaminant. All statistically significant correlations between OC concentrations and lymphocyte proliferation had positive slopes, indicating that turtles with higher contaminant levels exhibited elevated lymphocyte proliferation responses (Figures 2 and 3). Correlations were also examined between lymphocyte proliferation and OC concentrations calculated on a lipid-normalized basis (data not shown). These correlations were very similar to those shown in Table 1.

### *Lymphocyte proliferation:* in vitro *exposure experiments.*

PBLs from 16 turtles were exposed to increasing concentrations of Aroclor 1254 (0–13.5 μg/mL) or 4,4′-DDE (0–13.4 μg/mL). Cell viability was measured for 8 of these turtles after 5 days of contaminant exposure, examining only cells not stimulated with the mitogen (data not shown). Viability was unaffected by concentrations of ≤ 1,000 ng/mL of either contaminant. Both contaminants significantly decreased cell viability at concentrations of ≥ 7,500 ng/mL. Therefore, lymphocyte proliferation responses at these higher concentrations are not reported.

**Figure 4:**
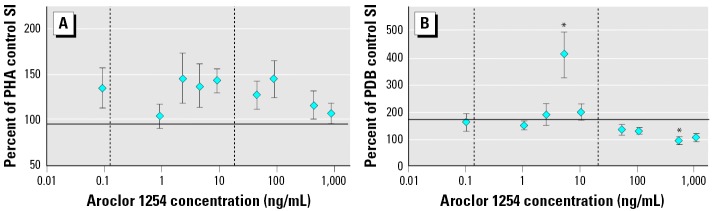
The effect of a 5-day *in vitro* exposure to Aroclor 1254 on loggerhead sea turtle lymphocyte proliferation (SI) responses stimulated by 5 μg/mL PHA (*A*) and 0.2 μg/mL PDB (*B*). Data are shown as mean ± SE of the percentage of the SI measured in the control (no DMSO or Aroclor 1254) for each turtle. Sample sizes are 8 or 16 depending on the treatment group. The *x*-axis crosses the *y*-axis at the percentage of the control value for the wells receiving only DMSO. The mean ± SE SI for the DMSO controls in the PHA and PDB experiments was 96.6 ± 15.0 and 172 ± 39, respectively. Vertical dashed lines indicate the range of ∑PCB concentrations measured in the blood of 17 loggerhead sea turtles used in the correlative field study. *Significantly different from the DMSO control (ANOVA with log-transformed data, Dunnet’s multiple comparison test; *p* < 0.05).

The effects of *in vitro* exposure to Aroclor 1254 on lymphocyte proliferation responses are shown in Figure 4. All of the tested concentrations of Aroclor 1254 generally increased PHA-induced proliferation, albeit not significantly, compared with the response in the DMSO control (Figure 4A). *In vitro* exposure to 5 ng/mL Aroclor 1254 significantly increased PDB-induced proliferation, whereas 498 ng/mL Aroclor 1254 significantly decreased this response (Figure 4B).

**Figure 5:**
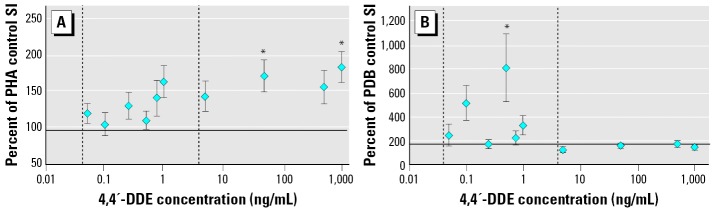
The effect of a 5-day *in vitro* exposure to 4,4′-DDE on loggerhead sea turtle lymphocyte proliferation (SI) responses stimulated by 5 μg/mL PHA (*A*) and 0.2 μg/mL PDB (*B*). Data are shown as mean ± SE of the percentage of the SI measured in the control (no DMSO or 4,4′-DDE) for each turtle. Sample sizes are 8 or 16 depending on the treatment group. The *x*-axis crosses the *y*-axis at the percentage of the control value for the wells receiving only DMSO. The mean ± SE SI for the DMSO controls in the PHA and PDB experiments was 96.6 ± 15.0 and 172 ± 39, respectively. Vertical dashed lines indicate the range of 4,4′-DDE concentrations measured in the blood of 17 loggerhead sea turtles used in the correlative field study. *Significantly different from the DMSO control (ANOVA with log-transformed data, Dunnet’s multiple comparison test; *p* < 0.05).

*In vitro* exposure to 4,4′-DDE significantly enhanced PHA- and PDB-induced proliferation (Figure 5). Increasing concentrations of 4,4′-DDE administered to the culture wells produced an increasing trend in PHA-induced proliferation (Figure 5A). Statistically significant increases from the DMSO control were observed at 48 ng/mL and 992 ng/mL 4,4′-DDE. Only one concentration of 4,4′-DDE (0.5 ng/mL) caused a statistically significant effect on PDB-induced proliferation (Figure 5B). Additionally, it should be noted that B-lymphocyte proliferation appeared to be more sensitive than did T-lymphocyte proliferation. Statistically significant effects on PDB-induced proliferation were observed at lower concentrations of both contaminants compared with PHA-induced proliferation (Figures 4 and 5).

## Discussion

The field of wildlife immunotoxicology is relatively new, and studies examining reptiles have been initiated only in the last 10 years (reviewed by Keller et al., 2006). The present study is the first published study to demonstrate that OCs may modulate immune function in sea turtles. Because the OC concentrations found in loggerhead turtles are lower than those observed to alter immunity in other species, we did not expect to observe significant correlations. The fact that even moderate correlations were observed with immune function suggests that loggerhead sea turtles may be sensitive to the immunomodulatory effects of OCs.

One concern regarding correlative studies is interaction with other factors, such as sex, body condition, or hormone concentrations. The latter may be of significant importance when dealing with endocrine-active compounds such as OCs. Other studies assessing loggerhead turtles from South Carolina, Georgia, and Florida have reported that testosterone levels in juvenile female loggerhead turtles weakly and negatively correlate with PDB-induced lymphocyte proliferation (*n* = 127), but body condition and sex are not related to any of the proliferation responses (Keller et al. 2005). Previous analyses of the present data set showed that OC concentrations were not significantly different between sexes, and they did not correlate with body condition (Keller et al. 2004a, 2004c). OC concentrations did not correlate with testosterone levels in this data set, either (*n* = 48; *p* > 0.05). Estrogen levels were not measured because plasma estrogen levels in juvenile sea turtles are typically below detection limits (Owens 1997). We observed no relationships with body condition, sex, or testosterone in the present data set for lymphocyte proliferation or lysozyme activity, except for one significant, positive correlation between body condition and proliferation stimulated with 0.2 μg/mL PDB. However, this particular mitogen and media type did not correlate with plasma OC concentrations. The overall lack of interaction of these additional variables adds further strength to the argument that OC concentrations may be modulating immune measurements and that the observed effects are not related to a covarying, confounding factor.

Another recognized limitation of this study is the use of multiple individual correlations. Using this statistical approach, we expected 5% of correlations to be significant by chance alone, which is 3 of the 45 correlations in Table 1. Half of those would be expected to have positive slopes, whereas the other half would be negative. Because we observed 6 significant correlations and all had positive slopes, we conclude that these correlations were not likely due to chance alone. This correlative study, designed as a pilot study to investigate possible contaminant effects, led to a hypothesis that OCs may enhance lymphocyte proliferation. This hypothesis was subsequently tested in the *in vitro* component of this study.

All of the statistically significant correlations between OC concentrations and lymphocyte proliferation were positive, suggesting immunoenhancement (Table 1). Although traditional thought would have expected immune suppression (Harper et al. 1993; Silkworth et al. 1984), enhanced lymphocyte proliferation and PHA skin responses have been observed after OC exposure in several controlled laboratory studies with wildlife species (Peden-Adams 1999; Segre et al. 2002; Smits et al. 2002; Wu et al. 1999). Environmental studies with free-ranging wildlife and epidemiologic studies with humans have also shown significant, positive relationships between lymphocyte proliferation and OC exposure (Croisant and Grasman 2002; Lü and Wu 1985; Peden-Adams et al. 1996; Rooney et al. 2003). The immunoenhancement noted in these previous laboratory and environmental studies support the conclusions that OCs may enhance certain responses of loggerhead turtle immune cells and that lymphocyte proliferation may indeed be a useful biomarker of exposure to OCs. It should be noted, however, that enhancement of immune responses is not necessarily a healthy outcome, because immunoenhancement can lead to autoimmune diseases and hypersensitivity (Burns et al. 1996). Any alteration of immune function, even enhancement, can be considered an adverse effect.

Admittedly, results from correlative field studies are largely circumstantial, and no causal relationship can be identified with certainty. Because intentional, experimental exposure of protected sea turtles to contaminants is not feasible, we used *in vitro* experiments to further investigate the immunoenhancement suggested by the correlations observed between lymphocyte proliferation and actual environmental exposure to OCs in the pilot study. Although typical dose–response curves were not always observed in the *in vitro* experiments, similarities between these experiments and the correlative field study were seen not only in the concentrations that produced significant results but also, more consistently, in the direction of the response. Both study components demonstrated immunoenhancement instead of suppression. Interestingly, *in vitro* exposure to both PCBs and 4,4′-DDE significantly enhanced PDB-induced proliferation at concentrations that are found in loggerhead sea turtle blood (vertical lines in Figures 4B and 5B). Specifically, *in vitro* exposure to 5 ng/mL Aroclor 1254 significantly increased PDB-induced proliferation (Figure 4B), and the two turtles with the strongest PDB-induced proliferation responses in the correlative field study had blood ∑PCB concentrations similar to this (4.32 and 8.40 ng/g wet mass). Likewise, the one concentration of 4,4′-DDE (0.5 ng/mL) that increased PDB-induced proliferation responses *in vitro* (Figure 5B) is similar to the blood 4,4′-DDE concentrations measured in the top five responding turtles (0.603–1.13 ng/g wet mass) in the field study. Although these comparisons are not identical in methods (different PDB concentrations), these results, together with the general enhancement observed in Figures 4A and 5A, support the hypothesis that environmentally relevant concentrations of OCs may enhance certain loggerhead sea turtle immune responses.

Previous studies have examined similar *in vitro* exposures of mammalian lymphocytes to PCBs (De Guise et al. 1998; Smithwick et al. 2003; Snyder and Valle 1991). Only one of these studies, however, tested PCB concentrations in the same range as used in the present study (Snyder and Valle 1991). Snyder and Valle (1991) also observed immunoenhancement of lymphocyte proliferation using rat splenocytes exposed to 0.01 μg/mL Aroclor 1254. The other two studies demonstrated immunosuppression, but higher exposure concentrations were used (15 μg/mL of three PCB congeners combined, De Guise et al. 1998; 10 μg/mL Aroclor 1242, Smithwick et al. 2003). These concentrations are near the high end of the concentration range used in the present study that resulted in decreased cell viability. These comparisons suggest that stimulation of lymphocyte proliferation may occur at low levels of exposure and that sea turtle lymphocytes may be more sensitive to the cytotoxic effects of OCs than are mammalian cells.

To our knowledge, no previous study examining sea turtle immunity has measured innate (nonspecific) immune functions. Circulating lysozyme is a marker of pro-inflammatory responses, has antibacterial functions, and is a measure of innate immunity (Burton et al. 2002; Weeks et al. 1992). In mammals and fish, lysozyme is secreted by neutrophils (cellular equivalents of reptilian heterophils) upon entry of foreign bacteria and lyses gram-positive bacterial cells by degrading the cell wall (Balfry and Iwama 2004; Ito et al. 1997). In fish, PCBs are known to exhibit varied effects on lysozyme activity (Burton et al. 2002; Hutchinson et al. 2003). In the present study, we observed significant negative correlations between lysozyme and both 4,4′-DDE and ∑chlordanes. These findings suggest that OCs in the blood may suppress lysozyme production or activity in sea turtles. More recently, we have also assessed lysozyme activity in a separate set of juvenile loggerhead turtles captured from near shore waters of South Carolina, Georgia, and Florida and obtained similar results (Peden-Adams MM, Keller JM, unpublished data). In that study, blood ∑PCB, 4,4′-DDE, and ∑chlordane concentrations correlated with lysozyme activity (*r*_S_ = −0.496, −0.505, and −0.381, respectively, with *p* = 0.005, 0.004, and 0.038, respectively) measured in 30 turtles captured in July 2001. Interestingly, in that study and the present one, 4,4′-DDE exhibited very similar, statistically significant correlations. ∑DDT concentrations in human breast milk have been previously noted to correlate with lysozyme in milk (Saleh et al. 1998). Although the mechanism of decreased lysozyme is not understood, the data suggest that heterophil function is suppressed in turtles as exposure to OCs increases. Future studies should assess phagocytosis and respiratory burst to further elucidate effects of OCs on innate immune functions in sea turtles.

In a parallel study with the same sample set of loggerhead sea turtles, the ratio of heterophils to lymphocytes was significantly and positively correlated with adipose concentrations of mirex and dioxin-like PCBs (Keller et al. 2004c). An elevation in this ratio is a common response to many stressors in birds, mammals, and sea turtles (Aguirre et al. 1995; Grasman et al. 1996; Gross and Siegel 1983; Maxwell and Robertson 1998). The fact that concentrations of various OC classes in the loggerhead sea turtles were significantly correlated to four immune parameters (increased T-cell proliferation, increased B-cell proliferation, decreased lysozyme activity, and increased heterophil:lymphocyte ratio) provides additional evidence that turtles with elevated OC exposure exhibit immunomodulation.

## Conclusion

Certain lymphocyte proliferation responses in loggerhead sea turtles are positively correlated with OC concentrations measured in blood, even though the concentrations in sea turtles are generally much lower than in fish-eating wildlife. *In vitro* exposure experiments using relevant concentrations of both PCBs and 4,4′-DDE support the correlative field observations. Another measure of immune function, lysozyme activity, is also significantly correlated with concentrations of two major classes of OCs in the blood. These results are similar to the findings of many other wildlife studies and suggest that the sea turtle immune system is modulated by environmentally relevant concentrations of OCs. Future studies could use these relatively simple immune function assays as biomonitoring tools. They should also develop and optimize additional assays, such as natural killer cell activity, in order to more completely assess the effects of environmental contaminants on the immune system of sea turtles.

## Figures and Tables

**Table 1:** Mitogen-induced lymphocyte proliferation of loggerhead sea turtles and Spearman rank correlations between lymphocyte proliferation responses and OC concentration measured in whole blood.

	Mitogen					Spearman correlation coefficient [*r*_S_ (*p*-value)] between lymphocyte proliferation and OCs in whole blood		
Medium type^a^	Type^b^	Concentration (μg/mL)	Day^c^	Mean SI (SE)^d^	Sample size	∑PCBs	4,4′-DDE	∑Chlordanes
1	ConA (C5275)	20	4	3.94 (0.95)	19	−0.132 (0.591)	0.035 (0.989)	−0.065 (0.792)
	ConA (C5275)	20	5	2.47 (0.52)	24	−0.073 (0.735)	−0.020 (0.926)	−0.084 (0.698)
	LPS (L2630)	10	4	3.41 (0.52)	19	0.528 (0.020)*	0.495 (0.031)*	0.484 (0.036)*
	LPS (L2630)	10	5	3.01 (0.47)	24	0.249 (0.241)	0.170 (0.426)	0.157 (0.463)
	PDB (P1269)	0.2	4	4.52 (1.23)	19	0.074 (0.764)	−0.063 (0.797)	0.035 (0.887)
	PDB (P1269)	0.2	5	2.84 (0.55)	24	0.113 (0.600)	0.121 (0.574)	0.159 (0.459)
2	PHA	5	5	114 (55)	17	0.596 (0.012)*	0.431 (0.084)	0.434 (0.082)
	PHA	10	5	29.1 (13.5)	17	0.020 (0.941)	0.020 (0.941)	0.003 (0.993)
	ConA (C2010)	10	5	1.89 (0.37)	10	0.370 (0.293)	0.370 (0.293)	0.406 (0.244)
	ConA (C2010)	20	5	3.56 (0.70)	24	−0.111 (0.605)	−0.118 (0.582)	−0.151 (0.480)
	LPS (L3129)	2.5	5	2.01 (0.39)	17	0.385 (0.127)	0.201 (0.439)	0.243 (0.348)
	LPS (L3129)	5	5	1.40 (0.25)	17	0.186 (0.474)	0.103 (0.694)	0.226 (0.384)
	PDB (P1269)	0.2	5	7.12 (2.88)	10	0.006 (0.987)	0.006 (0.987)	0.079 (0.829)
	PDB (P1269)	0.4	5	6.08 (1.73)	17	0.015 (0.955)	−0.005 (0.985)	0.012 (0.963)
	PDB (P1269)	0.8	5	4.10 (1.28)	17	0.564 (0.018)*	0.507 (0.038)*	0.476 (0.054)

a Media types are described in “Materials and Methods” and differ only by the manufacturer of FBS.

b Catalog numbers for mitogens purchased from Sigma are shown in parentheses; PHA was purchased from Amersham Pharmacia Biotech.

c Duration of mitogen exposure indicating when ^3^H-thymidine was added (96 hr or 120 hr, respectively).

d SI = cpm of mitogen-stimulated cells/cpm of unstimulated cells. Data from Keller et al. (2005). *p < 0.05.
